# Identification of subclasses of sepsis that showed different clinical outcomes and responses to amount of fluid resuscitation: a latent profile analysis

**DOI:** 10.1186/s13054-018-2279-3

**Published:** 2018-12-18

**Authors:** Zhongheng Zhang, Gensheng Zhang, Hemant Goyal, Lei Mo, Yucai Hong

**Affiliations:** 1 Department of Emergency Medicine, Sir Run Run Shaw Hospital, Zhejiang University School of Medicine, No. 3, East Qingchun Road, Hangzhou, 310016 Zhejiang Province China; 2 Department of Critical Care Medicine, Second Affiliated Hospital, Zhejiang University School of Medicine, Hangzhou, 310009 Zhejiang China; 3 Department of Internal Medicine, Mercer University School of Medicine, Macon, GA 31201 USA; 4 Department of Biostatistics, Lejiu Healthcare Technology Co., Ltd, Shanghai, China

**Keywords:** Sepsis, Mortality, Latent profile analysis, Subclass, Fluid response

## Abstract

**Background and objective:**

Sepsis is a heterogeneous disease and identification of its subclasses may facilitate and optimize clinical management. This study aimed to identify subclasses of sepsis and its responses to different amounts of fluid resuscitation.

**Methods:**

This was a retrospective study conducted in an intensive care unit at a large tertiary care hospital. The patients fulfilling the diagnostic criteria of sepsis from June 1, 2001 to October 31, 2012 were included. Clinical and laboratory variables were used to perform the latent profile analysis (LPA). A multivariable logistic regression model was used to explore the independent association of fluid input and mortality outcome.

**Results:**

In total, 14,993 patients were included in the study. The LPA identified four subclasses of sepsis: profile 1 was characterized by the lowest mortality rate and having the largest proportion and was considered the baseline type; profile 2 was characterized by respiratory dysfunction; profile 3 was characterized by multiple organ dysfunction (kidney, coagulation, liver, and shock), and profile 4 was characterized by neurological dysfunction. Profile 3 showed the highest mortality rate (45.4%), followed by profile 4 (27.4%), 2 (18.2%), and 1 (16.9%). Overall, the amount of fluid needed for resuscitation was the largest on day 1 (median 5115 mL, interquartile range (IQR) 2662 to 8800 mL) and decreased rapidly on day 2 (median 2140 mL, IQR 900 to 3872 mL). Higher cumulative fluid input in the first 48 h was associated with reduced risk of hospital mortality for profile 3 (odds ratio (OR) 0.89, 95% CI 0.83 to 0.95 for each 1000 mL increase in fluid input) and with increased risk of death for profile 4 (OR 1.20, 95% CI 1.11 to 1.30).

**Conclusion:**

The study identified four subphenotypes of sepsis, which showed different mortality outcomes and responses to fluid resuscitation. Prospective trials are needed to validate our findings.

**Electronic supplementary material:**

The online version of this article (10.1186/s13054-018-2279-3) contains supplementary material, which is available to authorized users.

## Introduction

Sepsis is one of the leading causes of mortality and morbidity in the patients admitted to intensive care units (ICU). Despite evolving concepts and advances in management, the mortality associated with sepsis remains unexpectedly high. Many large clinical trials have been conducted aiming to test whether any drugs (for example, corticosteroids and ulinastatin) or other interventions (such as early goal-directed therapy, fluid strategy) could reduce the mortality but have yielded conflicting results [1–6]. One of the possible reasons that these sepsis trials failed to identify positive results was the problem of the case mix. Sepsis encompasses a heterogeneous population with respect to the site of infection, type of organism, genetic background, and coexisting conditions of the host. Thus, it is recommended that the individualized patient care be mandatory to improve survival outcome [7]. The concept of individualized medicine is to identify subphenotypes of patients who present with distinct clinical characteristics and respond to personalized interventions. For example, Calfee et al. identified subphenotypes of acute respiratory distress syndrome which showed distinct clinical characteristics [8]. In sepsis, many efforts have been made to identify endotypes by using genomics and transcriptomics [9]. However, genotyping is not routinely performed in daily clinical practice and thus such practice remains in the research stage. It is also suggested that individualized fluid strategy be implemented for sepsis [10]. However, there is a lack of empirical evidence on how to individualize patients with sepsis on the basis of clinical variables. The study aimed to analyze data to see whether subgroups could be detected. Electronic health-care records (EHRs) were employed for the study; the indicator variables for building a latent profile model were readily available in daily clinical practice. Furthermore, the identified profiles were compared for their different responses to fluid input.

## Methods

### Critical care database

The critical care big data Medical Information Mart for Intensive Care (MIMIC-III) was employed for this study. MIMIC-III is a large, single-center database comprising information relating to patients admitted to ICUs at a large tertiary care hospital [11, 12]. MIMIC-III integrates de-identified, comprehensive clinical data of patients admitted to the Beth Israel Deaconess Medical Center in Boston, MA, USA, from June 1, 2001 to October 31, 2012. There were 53,423 distinct hospital admissions for adult patients (16 years or above) admitted to ICUs during the study period [11]. This study was an analysis of the third-party anonymized databases with pre-existing institutional review board (IRB) approval; thus, IRB approval from our institution was exempted.

### Study population

In the third sepsis definition, sepsis was defined as life-threatening organ dysfunction caused by a dysregulated host response to infection [13]. In this study, we screened patients with documented or suspected infection, plus the presence of organ dysfunction [14, 15]. The International Classification of Diseases, Ninth Revision, Clinical Modification (ICD-9-CM) codes for a bacterial or fungal infection were used to define infection (Additional file 1). A patient was defined to have organ dysfunction if he or she had ICD-9 code as follows: unspecified thrombocytopenia (287.5), hypotension (458.9), acute and subacute necrosis of liver (570), acute kidney failure (584.9), anoxic brain damage (348.1), shock without mention of trauma (785.59), encephalopathy (348.30), transient mental disorders due to conditions classified elsewhere (293.9), secondary thrombocytopenia (287.49), other and unspecified coagulation defects (286.9), defibrination syndrome (286.6), and hepatic infarction (573.4). If mechanical ventilation (procedures ICD code: 96.70, 96.71, 96.72) was required, it was also defined as organ dysfunction. The method was adapted from Angus DC [16], and the Structured Query Language (SQL) code could be found at https://github.com/MIT-LCP.

Multiple hospital admissions from the same patient were included as independent cases. Only the first ICU admission was included for analysis for patients who had multiple admissions to ICU.

### Demographical and laboratory variables

The following variables were extracted from the MIMIC- III database: age at the time of hospital admission, gender, admission type, sequential organ failure assessment (SOFA) score, each component of SOFA score, use of vasopressors (including dopamine, epinephrine, norepinephrine, phenylephrine, and vasopressin), and renal replacement therapy (RRT). SOFA score was calculated within the first 24 h after the ICU admission. The laboratory variables included platelet count, activated partial thrombin time (aPTT), international normalized ratio (INR), and creatinine. Other clinical variables such as urine output (UO) for the first 24 h, Glasgow Coma Scale (GCS) score, mean blood pressure (BP), vasopressors, and arterial partial oxygen pressure (PaO_2_) were included. The median value was computed for variables measured more than once during the first 24 h after ICU admission. The lowest value of GCS score reported in the first 24 h was used in the study.

The primary endpoint was hospital mortality, which was defined as the status of patient survival at the time of hospital discharge. Secondary endpoints included length of stay (LOS) in the ICU and hospital. The 90-day mortality was also obtained by linking to the social security database by the database investigators.

### Missing values

Variables with more than 40% missing values were excluded from the analysis (Additional file 1: Figure S1). Variables such as base excess, albumin, and calcium had missing values greater than 40% and were excluded from the study. Multiple imputation was performed for the remaining variables [17].

### Latent profile analysis

Clinical variables were selected for constructing latent profiles as indicator variables. Platelet count, aPTT, and INR were used for the hematological system; creatinine and UO were used for the renal function; GCS score was used to assess the cerebral function, the circulatory system was measured by the mean BP and vasopressors, and respiratory function was measured by PaO_2_ and partial pressure of carbon dioxide (PaCO_2_). Multiple kinds of vasopressors (including dopamine, epinephrine, norepinephrine, phenylephrine, and vasopressin) were recorded in the MIMIC-III database. Therefore, their use was scaled by the standard deviation and centered at mean and then combined as one variable. Continuous variables were scaled to have similar variances. The distributions of included variables were examined before analysis, and severely skewed data would be transformed.

The goal of latent profile analysis (LPA) is to fit a mixture of distributions. The keys are that the underlying distributions must exist and the analyst must have the variables that best separate out those distributions [18–20]. In the study, the number of profiles was determined by Bayesian information criteria (BIC) and bootstrap likelihood ratio test (BLRT). Specifically, BIC was used to compare models with different numbers of clusters or specifying different parameterizations or both. Lower values of the BIC are indicative of better model fit. BLRT was used to assess the number of mixture components in a specific finite mixture model parameterization. The observed significance is approximated by using the bootstrap for the likelihood ratio test statistic (LRTS). BLRT computed *P* values for the comparison of k-class model with (k-1)-class model [21]. A *P* value of 0.05 was used to judge the statistical significance for the bootstrap likelihood ratio test. Furthermore, because the number of patients should be sizable in each latent profile, we pre-specified that the patient proportion should be greater than 5% in any of the latent profiles [22]. The clinical interpretation was also considered when determining the number of latent profiles. The LPA model was first fit by using patients admitted before 2008 and then validated in patients admitted after 2008. The final LPA model was fit on the whole dataset.

### Statistical analysis

Continuous variables were expressed as the mean (standard deviation) or median (interquartile range, or IQR) as appropriate and were compared between the different profiles of sepsis using analysis of variance [23]. The CBCgrps package was employed for the statistical description and bivariate inference [24]. Clinical outcomes such as the mortality rate, LOS in the ICU, and the entire hospitalization were compared between latent profiles.

The multivariable logistic regression model was employed to investigate the independent association of fluid input and mortality outcome, and an interaction between fluid input and latent profiles was included. Other covariates included in the models were SOFA score, age, gender, admission type, ethnicity, ICU types, and the use of RRT. The covariates were selected because they were potential confounders as determined by subject-matter knowledge. Odds ratio (OR) and relevant 95% confidence interval (CI) were reported for the impact of each 2000 mL increase in fluid input on mortality outcome.

All statistical analyses were performed by using R package (version 3.4.3). A *P* value less than 0.05 was considered to be statistically significant.

## Results

### Choose the number of latent classes

A total of 14,993 patients fulfilled the inclusion criteria for the analysis. Models with different number of profiles were compared. In patients enrolled before 2008, the best number of profiles was 4, which was validated in patients admitted after 2008 (Additional file 1: Tables S1 and S2). Then the LPA model was fit to the whole dataset. The BIC and AIC values decreased from 3-class model rapidly to the 4-profile model (dropped by 10,000) and remained relatively stable from 4- to 5-profile (decreased by 3000). The entropy dropped remarkably from 4- to 5-profile model. The number of patients in each profile was less than 5% for the 6- and 7-profile models. Taken together, the 4-profile model was chosen as the best one (Table 1).

**Table 1:** Latent profile analysis for choosing the best number of profiles

						Number of patients in each latent profile							
Number of classes	AIC	CAIC	BIC	SABIC	Entropy	1	2	3	4	5	6	7	*P* value
2	2,254,774.736	2,255,429.502	2,255,353.502	2,255,111.98	0.975	12,865 (86)	2128 (14)						0.001
3	2,244,946.905	2,245,825.67	2,245,723.67	2,245,399.522	0.955	11,525 (77)	1580 (11)	1888 (13)					0.001
4	2,235,891.639	2,236,994.403	2,236,866.403	2,236,459.629	0.953	10,340 (69)	1305 (9)	1635 (11)	1713 (11)				0.001
5	2,232,244.359	2,233,571.121	2,233,417.121	2,232,927.722	0.887	8010 (53)	1188 (8)	2561 (17)	1552 (10)	1682 (11)			0.063
6	2,228,493.639	2,230,044.4	2,229,864.4	2,229,292.374	0.886	7586 (51)	1200 (8)	2367 (16)	1476 (10)	1599 (11)	765 (5)		0.075
7	2,223,495.867	2,225,270.627	2,225,064.627	2,224,409.976	0.887	7108 (47)	1080 (7)	2272 (15)	1293 (9)	1051 (7)	1505 (10)	684 (5)	0.081

*P* value was reported for the bootstrap likelihood ratio test comparing the current model (k class) to the model with k-1 class.

*Abbreviations*: *AIC* Akaike information criterion, *BIC* Bayesian information criteria, *CAIC* consistent Akaike information criterion, *SABIC* sample size adjusted Bayesian information criteria

### Different clinical features between profiles

**Fig. 1:**
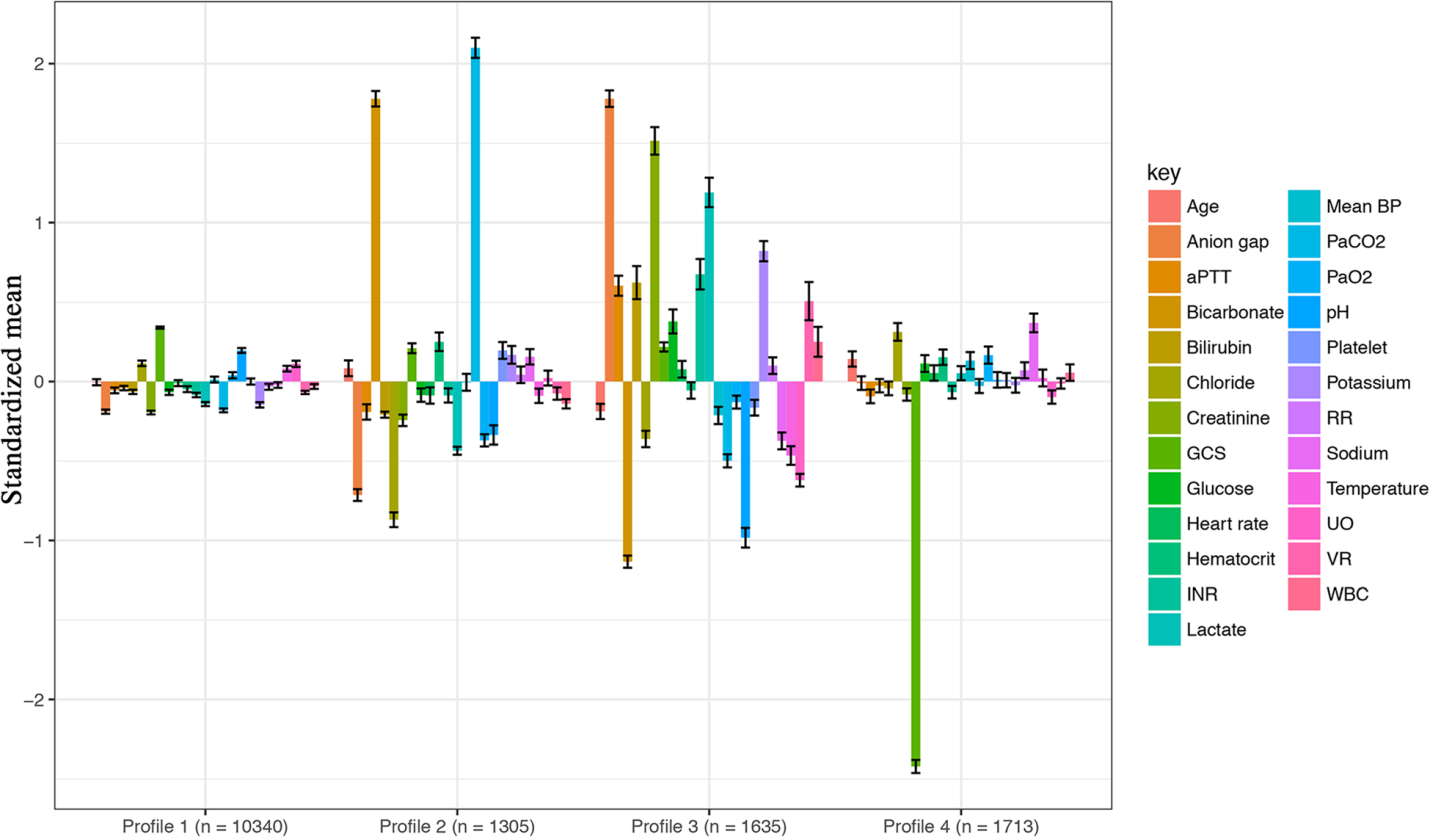
Characteristics of latent profile groups. The y-axis shows the standardized mean for each variable (that is, each variable is centered at the sample mean and scaled by its standard deviation). Abbreviations: *aPTT* activated partial thrombin time, *BP* blood pressure, *GCS* Glasgow Coma Scale, *INR* international normalized ratio, *RR* respiratory rate, *UO* urine output, *VR* vasopressor rate, *WBC* white blood cell count

Clinical features of all the four profiles are shown in Tables 2 and 3. Profile 1 (69%) was the largest group and had the lowest mortality rate (16.9%) and was considered the baseline type; profile 2 (9%) was characterized by respiratory dysfunction (low PaO_2_ and high PaCO_2_); profile 3 (11%) was characterized by multiple organ dysfunction (kidney, coagulation, liver, and shock); and profile 4 (11%) was characterized by neurological dysfunction (low GCS score) (Fig. 1). Table 2 shows that profile 3 has the largest amount of vasopressor use and the lowest BP.

**Table 2:** Continuous variables included in the mixture modeling

Clinical variables, mean (SD) or median (IQR)	Profile 1 (*n* = 10,340)	Profile 2 (*n* = 1305)	Profile 3 (*n* = 1635)	Profile 4 (*n* = 1713)	*P* values
Age, years	66.81 (16.47)	68.25 (15.00)	63.79 (16.23)	69.21 (16.64)	<0.001
Ph	7.39 (0.06)	7.35 (0.09)	7.29 (0.10)	7.37 (0.08)	<0.001
Bicarbonate, mmol/L	22.92 (3.75)	32.48 (4.73)	17.19 (4.17)	23.00 (4.66)	<0.001
Hematocrit, %	31.61 (5.27)	33.24 (5.89)	31.56 (5.85)	32.71 (5.60)	<0.001
Platelet, ×10^9^/L	208.00 [140.50, 288.00]	233.50 [171.00, 310.50]	183.00 [109.25, 278.00]	208.00 [146.00, 287.50]	<0.001
aPTT, s	38.14 (18.10)	35.51 (17.29)	50.91 (25.18)	37.42 (17.73)	<0.001
INR	1.57 (0.88)	1.57 (0.98)	2.48 (2.36)	1.59 (0.99)	<0.001
Creatinine, mg/dL	1.44 (1.02)	1.36 (1.09)	4.32 (3.01)	1.64 (1.38)	<0.001
Urine output, L/24 h	1.54 [0.93, 2.42]	1.48 [0.94, 2.23]	0.50 [0.14, 1.14]	1.32 [0.73, 2.12]	<0.001
Minimum GCS score	15.00 [14.00, 15.00]	15.00 [14.00, 15.00]	15.00 [14.00, 15.00]	7.00 [4.00, 9.00]	<0.001
Bilirubin, mg/dL	0.60 [0.40, 1.15]	0.50 [0.30, 0.80]	0.90 [0.40, 2.88]	0.60 [0.40, 1.10]	<0.001
Mean BP, mm Hg	80.44 (16.47)	80.15 (16.85)	76.57 (18.84)	82.50 (19.39)	<0.001
Vasopressor rate^a^	0.08 (0.63)	0.07 (0.85)	0.77 (2.96)	0.15 (0.82)	<0.001
PaO_2_, mm Hg	136.25 (69.02)	108.53 (48.23)	124.77 (57.29)	144.90 (77.48)	<0.001
PaCO_2_, mm Hg	38.75 (6.79)	63.57 (12.71)	35.31 (9.30)	40.42 (10.15)	<0.001
Lactate, mmol/L	2.13 (1.20)	1.59 (0.85)	4.57 (3.50)	2.49 (1.73)	<0.001
Temperature, °C	36.90 (0.72)	36.77 (0.64)	36.48 (0.93)	36.85 (0.85)	<0.001
Glucose, mg/dL	144.77 (54.97)	143.63 (49.28)	173.07 (98.95)	156.27 (70.68)	<0.001
Anion gap, mEq/L	14.37 (2.77)	12.26 (2.76)	22.32 (4.33)	15.10 (3.65)	<0.001
Chloride, mmol/L	105.25 (5.96)	98.70 (5.68)	102.09 (7.10)	106.57 (7.85)	<0.001
WBC, ×10^9^/L	12.92 (9.20)	11.62 (6.37)	16.25 (22.96)	13.95 (12.96)	<0.001
Sodium, mmol/L	138.25 (4.93)	139.20 (4.79)	136.38 (5.85)	140.34 (6.63)	<0.001
Potassium, mmol/L	4.15 (0.59)	4.37 (0.70)	4.81 (0.88)	4.24 (0.67)	<0.001
Heart rate, /min	90.35 (16.82)	89.02 (16.11)	91.84 (18.48)	91.44 (17.52)	<0.001
Respiratory rate, /min	20.66 (4.44)	21.00 (4.35)	21.26 (4.90)	21.13 (4.82)	<0.001

^a^Vasopressors included dopamine and norepinephrine. Since the dosings of these drugs were in different scale, they were centered at mean and scaled by standard deviation and combined, referring to as vasopressors. For patients without using vasopressor, the value was 0.

*Abbreviations*: *aPTT* activated partial thrombin time, *BP* blood pressure, *GCS* Glasgow Coma Scale, *INR* international normalized ratio, *IQR* interquartile ratio, *PaCO*_*2*_ partial pressure of carbon dioxide, *PaO*_*2*_ arterial partial oxygen pressure, *pH* potential hydrogen, *SD* standard deviation, *WBC* white blood cell count

**Table 3:** Categorical variables and outcome/treatment variables not included in the mixture modeling

Clinical variables, *n* (%)	Profile 1 (*n* = 10,340)	Profile 2 (*n* = 1305)	Profile 3 (*n* = 1635)	Profile 4 (*n* = 1713)	*P* values
Male gender	5539 (53.6)	601 (46.1)	943 (57.7)	884 (51.6)	<0.001
Ethnicity					<0.001
Asian	246 (2.4)	19 (1.5)	45 (2.8)	64 (3.7)	
Black	879 (8.5)	156 (12.0)	269 (16.5)	216 (12.6)	
Hispanic	331 (3.2)	34 (2.6)	64 (3.9)	59 (3.4)	
Unknown	1217 (11.8)	126 (9.7)	186 (11.4)	184 (10.7)	
White	7667 (74.1)	970 (74.3)	1071 (65.5)	1190 (69.5)	
Admission type					<0.001
Elective	648 (6.3)	43 (3.3)	51 (3.1)	84 (4.9)	
Emergency	9438 (91.3)	1229 (94.2)	1546 (94.6)	1581 (92.3)	
Urgent	254 (2.5)	33 (2.5)	38 (2.3)	48 (2.8)	
Type of ICU					<0.001
CCU	1307 (12.6)	190 (14.6)	236 (14.4)	166 (9.7)	
CSRU	894 (8.6)	42 (3.2)	54 (3.3)	125 (7.3)	
MICU	5515 (53.3)	874 (67.0)	1071 (65.5)	972 (56.7)	
SICU	1515 (14.7)	123 (9.4)	201 (12.3)	309 (18.0)	
TSICU	1109 (10.7)	76 (5.8)	73 (4.5)	141 (8.2)	
D1 fluid input (median [IQR])	5115.43 [2661.98, 8800.37]	2624.94 [1320.00, 4925.19]	6047.19 [3001.75, 11,219.73]	5683.17 [3165.68, 9129.45]	<0.001
D2 fluid input (median [IQR])	2139.66 [900.00, 3871.71]	1638.55 [720.00, 2949.81]	2019.12 [592.79, 4435.06]	2690.00 [1209.69, 4589.09]	<0.001
Vasopressor, *n* (%)	2558 (24.7)	254 (19.5)	855 (52.3)	485 (28.3)	<0.001
SOFA (median [IQR])	4.00 [2.00, 6.00]	4.00 [3.00, 6.00]	9.00 [6.00, 12.00]	8.00 [5.00, 10.00]	<0.001
90-day mortality, *n* (%)	2358 (22.8)	346 (26.5)	744 (45.5)	557 (32.5)	<0.001
Hospital LOS (median [IQR])	12.00 [7.00, 20.00]	10.00 [6.00, 17.00]	10.00 [5.00, 19.00]	11.00 [6.00, 20.00]	<0.001
ICU LOS (median [IQR])	4.00 [2.00, 9.00]	4.00 [2.00, 9.00]	4.00 [2.00, 9.00]	4.00 [2.00, 10.00]	<0.001
Hospital mortality, *n* (%)	1744 (16.9)	238 (18.2)	743 (45.4)	470 (27.4)	<0.001
Use of RRT, *n* (%)	395 (3.8)	52 (4.0)	458 (28.0)	84 (4.9)	<0.001

*Abbreviations*: *BP* blood pressure, *CCU* coronary artery unit, *CSRU* cardiac surgery recovery unit, *GCS* Glasgow Coma Scale, *ICU* intensive care unit, *IQR* interquartile range, *LOS* length of stay, *MICU* medical intensive care unit, *RRT* renal replacement therapy, *SICU* surgical intensive care unit, *SOFA* sequential organ failure assessment, *TSICU* trauma-neuro intensive care unit, *UO* urine output

Profile 3 showed the highest mortality rate (45.4%), and profile 1 showed the lowest mortality rate (16.9%). Profile 1 showed the longest LOS in the hospital (*P* <0.001; Table 3). Profile 3 had the highest SOFA score on day 1 (median 9, IQR 6 to 12), followed by profile 4 (median 8, IQR 5 to 10). Profile 1 had the lowest SOFA score (median 4, IQR 2 to 6).

### Fluid input

Overall, the amount of fluid input was the largest on day 1 (median 5115 mL, IQR 2662 to 8800 mL) and decreased rapidly on day 2 (median 2140 mL, IQR 900 to 3872 mL). Patients in profile 2 received less fluid input than all other profiles, and those in the profile 3 received the largest amount of fluid input on day 1 (Table 3). In the multivariable regression model by adjusting for SOFA score, age, gender, admission type, ethnicity, ICU type, and the use of RRT, higher cumulative fluid input in the first 48 h was associated with reduced risk of hospital death for profile 3 (OR 0.89, 95% CI 0.83 to 0.95 for each 1000-mL increase in fluid input) and with increased risk of death for profile 4 (OR 1.20, 95% CI 1.11 to 1.30). Of note, more fluid inputs were associated with improved outcome in profile 3, which was consistent with the fact that this profile was characterized by circulatory shock (lowest mean BP and elevated requirement of vasopressor).

### Clinical outcomes

By using profile 1 as reference, profile 3 (OR 2.16, 95% CI 1.88 to 2.47) was associated with increased risk of hospital morality (Table 4). In the Cox regression model investigating independent predictors of 90-day survival (Additional file 1: Table S4), profile 2 (hazard ratio (HR) 1.15, 95% CI 1.02 to 1.28) and 3 (HR 1.79, 95% CI 1.63 to 1.97) showed increased risk of 90-day mortality as compared with profile 1.

**Table 4:** Association of fluid input and mortality outcome in different profiles

Variables	Odds ratio	Lower limit of 95% CI	Upper limit of 95% CI	*P* value
Age (each 10-year increase)	1.26	1.22	1.30	<0.001
SOFA	1.24	1.22	1.27	<0.001
ICU type (CCU as reference)				
CSRU	0.72	0.58	0.89	0.002
MICU	1.12	0.98	1.28	0.086
SICU	1.14	0.96	1.35	0.140
TSICU	1.02	0.84	1.24	0.840
RRT (yes as reference)	0.74	0.63	0.88	<0.001
Admission type (elective surgery as reference)				
Emergency	1.85	1.47	2.34	<0.001
Urgent	1.60	1.13	2.28	0.008
Gender (female as reference)	1.03	0.94	1.12	0.543
Ethnicity (Asian as reference)				
Black	0.84	0.61	1.15	0.265
Hispanic	0.86	0.58	1.27	0.451
Unknown	2.01	1.49	2.74	<0.001
White	1.22	0.93	1.64	0.163
Vasopressor use (yes as reference)	0.78	0.70	0.88	<0.001
Profile (1 as reference)				
Profile 2	1.15	0.96	1.37	0.123
Profile 3	2.16	1.88	2.47	<0.001
Profile 4	0.94	0.82	1.07	0.359
Interaction between profile and fluid input^a^				
1	0.99	0.96	1.02	0.615
2	0.94	0.82	1.06	0.318
3	0.89	0.83	0.95	0.046
4	1.20	1.11	1.30	<0.001

^a^Fluid input was the cumulative fluid input for the first 48 h after intensive care unit (ICU) admission. Odds ratio of mortality was reported for each 1000-mL increase in fluid input at each level of profiles. There was statistically significant interaction between profile and cumulative fluid input. To facilitate clinical interpretation, the effect sizes (odds ratio) of fluid input within each profile were reported

*Abbreviations*: *CCU* coronary artery unit, *CI* confidence interval, *CSRU* cardiac surgery recovery unit, *MICU* medical intensive care unit, *RRT* renal replacement therapy, *SICU* surgical intensive care unit, *SOFA* sequential organ failure assessment, *TSICU* trauma-neuro intensive care unit

### Sensitivity analysis

**Fig. 2:**
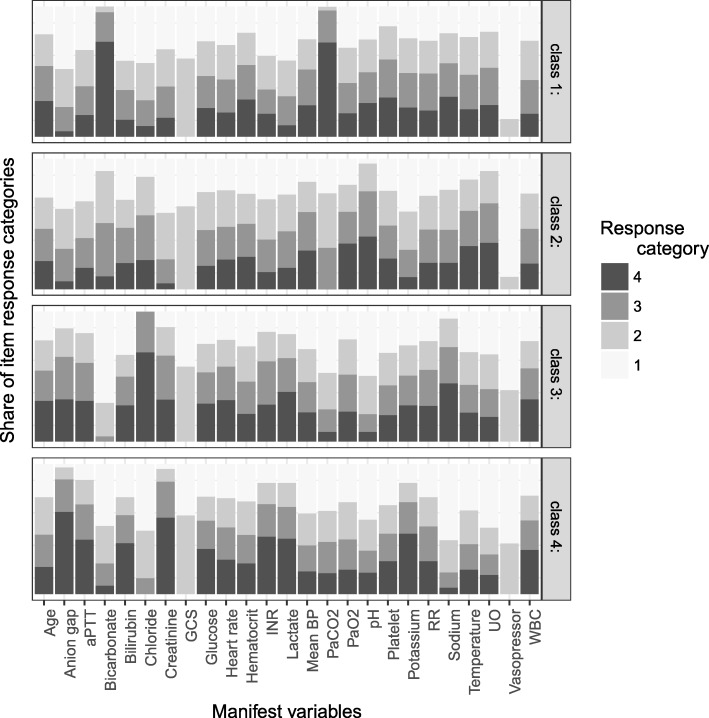
Characteristics of classes identified by latent class analysis. The response category of 1 to 4 is the quartile category by cutting continuous variables into four quartile categories. Category 1 refers to the lowest value and category 4 is the highest value. The vertical axis is the proportion of each response category. Abbreviations: *aPTT* activated partial thrombin time, *BP* blood pressure, *GCS* Glasgow Coma Scale, *INR* international normalized ratio, *RR* respiratory rate, *UO* urine output, *VR* vasopressor rate, *WBC* white blood cell count

It is of concern that the elevated aPTT in profile 3 might be explained by the use of heparin. Thus, sensitivity analysis was performed by restricting to patients without heparin (Additional file 1: Table S3). A subclass characterized by elevated aPTT and vasopressor requirement was identified (Additional file 1: Figure S2). The underlying subphenotypes were also verified by using latent class analysis. As shown in Additional file 1: Table S5, the best number of classes was 4 as judged by entropy. Characteristics of the four classes are shown in Fig. 2. Consistent with the result obtained by LPA, class 1 was characterized by respiratory dysfunction, class 2 was the baseline type, class 3 was characterized by neurological dysfunction, and class 4 was characterized by multiple organ dysfunction. Note that owing to the random process, the class number may not be consistent with the LPA model .

## Discussion

This study identified four subclasses of sepsis: profile 1 was the baseline group characterized by low mortality outcome; profile 2 was characterized by respiratory dysfunction; profile 3 was characterized by multiple organ dysfunction involving kidney, liver, coagulation, and circulatory failure; and profile 4 was characterized by neurological dysfunction. Whereas profile 1 showed the lowest mortality rate, profile 3 had the highest mortality rate. Since the profile 3 was characterized by hemodynamic instability, an increased amount of fluid input in the first two days was associated with improved mortality outcome, after adjustment for multiple confounding factors. For profile 4, more fluid input was associated with worse outcome.

The study employed LPA to identify subphenotypes of patients. There are a number of ways to perform agnostic clustering, such as latent variable mixture modeling, K-means clustering, and latent class analysis (LCA) [25–27]. Whereas LCA allows only categorical variables, the LPA allows continuous indicator variables. The main difference between LPA and other clustering algorithms is that LPA provides a “model-based clustering” approach that derives clusters using a probabilistic model that describes distribution of the data. So instead of looking for clusters with some arbitrary chosen distance measure, LPA fits a model that describes distribution of the data and based on this model you assess probabilities that certain patients are members of certain latent profiles. Because LPA uses a statistical model, assessing goodness of fit (GOF) is possible. The clustering method does not allow the assessment GOF. Furthermore, we had assumed that there were some processes or “latent structure” underlying the structure of our data. Thus, LPA seemed to be an appropriate choice since it allowed us to model the latent structure behind the data (rather than just looking for similarities).

Several studies have focused on the identification of subgroups of sepsis on the basis of the genomic and transcriptomic data [28–30]. In the pediatric septic shock, three subgroups were identified with subtype A showing a higher mortality rate, pediatric risk of mortality (PRISM) score, pediatric sepsis biomarker risk model (PERSEVERE)-based mortality risk, and maximum number of organ failures compared with other subtypes [31]. Sweeney et al. investigated subtypes of sepsis by using transcriptomics [32]. The study identified three subtypes of sepsis, which were coined “coagulopathic”, “adaptive”, and “inflammopathic” subtypes. In our study, coagulopathy was found to be present in profile 3, coexisting with other organ dysfunctions. Consistent with our study, the coagulopathic type showed the highest mortality rate. The adaptive subtype was equivalent to profile 1 in our study, which showed the best clinical outcome. However, transcriptomics are not routinely obtained in real clinical practice, which limited its widespread applicability. To the best of our knowledge, our study is the first to explore subphenotypes of the sepsis by using the clinical variables obtained from EHR, which would facilitate application of the results to daily clinical practice. Since the different subclasses showed different clinical presentations and responses to the fluid strategy, our sepsis classification could be used to design future trials. However, it is largely unknown whether tailored treatment according to the classification system is beneficial for patients with sepsis, and further trials are required to test this hypothesis.

Clinical variables employed for modeling LPA in the study included indicator variables for major organ dysfunctions that are typically involved in sepsis. The LPA model showed that the organ dysfunctions were related to each other with special patterns. For example, profile 3 was characterized by the multiple organ failure and circulatory shock, supporting the notion that multiple organ failures have common underlying pathophysiological process of circulatory shock. There is evidence that the circulatory shock usually coexists with coagulopathy and the latter is a good prognostic marker for survival outcome [33, 34]. Sepsis-associated encephalopathy (SAE) is an important complication of sepsis [35] and can occur in up to 82% of patients with sepsis [36]. SAE presents with varying severity ranging from mild confusion to coma. In this study, we identified a subgroup of sepsis patients who were characterized by the severe SAE with a median GCS score of 7 points. The mortality rate of this subclass was the second highest despite preserved functions of other organs, indicating the importance of neurological injury in sepsis.

Several limitations must be acknowledged in this study. First, this study used EHR data which were produced by routine clinical practice. Thus, having missing values is a big problem. Although there are many sophisticated methods to deal with missing values, significant bias may be introduced for those with missing rates greater than 40% [17, 37]. Thus, these variables were excluded from analysis. Multiple imputations were performed for variables containing missing values. Second, although restricting the variables used for modeling to those available in clinical practice is reasonable, it may limit the separation of classes. It would be better to use biomarkers and genomics as well. However, the biomarkers not routinely obtained were not available in the database. Third, the study was limited by the lack of external validation and thus the results need to be validated in other external datasets. However, we performed internal validation by splitting the whole dataset. The LPA model was first fit in patients enrolled before 2008 and then the model was validated in patients admitted after 2008. Fourth, fluid responsiveness was assessed observationally, as one of several exposures among several subgroups; hence, this association is less robust than if this were done within a randomized trial context. Finally, LPA does not provide a definitive class membership but instead provides posterior probabilities of each class and assigns the class with the highest posterior probability. Because of this, there is uncertainty regarding class membership (for example, patient A has 90% probability of being in class 1, 2% in class 2, 4% in class 3, and 4% in class 4).

## Conclusion

This study identified four subphenotypes of sepsis, which showed different mortality outcomes and responses to fluid resuscitation. The subphenotypes need to be validated in external datasets.

## Additional file

Additional file 1: **Figure S1.** Missing rate for clinical and laboratory variables extracted from the database. Variables with missing rate greater than 40% were excluded from analysis. **Figure S2.** Characteristics of latent profile groups by restricting to patients without using heparin. Owing to the random process, the specific profile number may not be consistent with the main analysis. Abbreviations: *aPTT* activated partial thrombin time, *BP* blood pressure, *GCS* Glasgow Coma Scale, *INR* international normalized ratio, *RR* respiratory rate, *UO* urine output, *VR* vasopressor rate, *WBC* white blood cell count. **Table S1.** Latent profile analysis restricting to patients admitted after 2008. **Table S2.** Latent profile analysis restricting to patients admitted before 2008. **Table S3.** Sensitivity analysis restricting to patients who did not receive heparin. **Table S4.** Cox regression model to adjust for confounding for the 90-day survival. **Table S5.** Choosing the best number of classes by using latent class analysis. (DOCX 76 kb)

## Abbreviations

aPTT: Activated partial thrombin time
BIC: Bayesian information criteria
BLRT: Bootstrap likelihood ratio test
BP: Blood pressure
CI: Confidence interval
EHR: Electronic health-care record
GCS: Glasgow Coma Scale
GOF: Goodness of fit
HR: Hazard ratio
ICD: International Classification of Diseases
ICU: Intensive care unit
INR: International normalized ratio
IQR: Interquartile range
IRB: Institutional review board
LCA: Latent class analysis
LOS: Length of stay
LPA: Latent profile analysis
MIMIC-III: Medical Information Mart for Intensive Care
OR: Odds ratio
PaCO_2_: Partial pressure of carbon dioxide
PaO_2_: Arterial partial oxygen pressure
RRT: Renal replacement therapy
SAE: Sepsis-associated encephalopathy
SOFA: Sequential organ failure assessment
UO: Urine output
