# Supplementary material for: Identification of subclasses of sepsis that showed different clinical outcomes and responses to amount of fluid resuscitation: a latent profile analysis
Source: Crit Care. 2018 Dec 18;22:347. doi: 10.1186/s13054-018-2279-3 (PMC6299613; doi:10.1186/s13054-018-2279-3)
Supplement: Supplementary file 1 — Figure S1. Missing rate for clinical and laboratory variables extracted from the database. Variables with missing rate greater than 40% were excluded from analysis. Figure S2. Characteristics of latent profile groups by restricting to patients without using heparin. Owing to the random process, the specific profile number may not be consistent with the main analysis. Abbreviations: aPTT activated partial thrombin time, BP blood pressure, GCS Glasgow Coma Scale, INR international normalized ratio, RR respiratory rate, UO urine output, VR vasopressor rate, WBC white blood cell count. Table S1. Latent profile analysis restricting to patients admitted after 2008. Table S2. Latent profile analysis restricting to patients admitted before 2008. Table S3. Sensitivity analysis restricting to patients who did not receive heparin. Table S4. Cox regression model to adjust for confounding for the 90-day survival. Table S5. Choosing the best number of classes by using latent class analysis. (DOCX 76 kb) [file 13054_2018_2279_MOESM1_ESM.docx]

Additional file to the article: Identification of subclasses of sepsis that showed different clinical outcomes and responses to amount of fluid resuscitation: a latent profile analysis

Zhongheng Zhang (MD),

Department of emergency medicine, Sir Run Run Shaw Hospital, Zhejiang University School of Medicine, Hangzhou, 310016, China.

Gensheng Zhang (MD；[genshengzhang@zju.edu.cn](mailto:genshengzhang@zju.edu.cn))

Department of Critical Care Medicine, Second Affiliated Hospital, Zhejiang University School of Medicine, Hangzhou, Zhejiang 310009, China.

Hemant Goyal (MD; [doc.hemant@yahoo.com](javascript:void(0))),

Department of Internal Medicine, Mercer University School of Medicine, Macon, GA USA 31201

Lei Mo (MPH; [lei.mo@le9health.com](mailto:lei.mo@le9health.com));

Department of biostatistics; Lejiu Healthcare Technology Co., Ltd

Yucai Hong (MD)

Department of emergency medicine, Sir Run Run Shaw Hospital, Zhejiang University School of Medicine, Hangzhou, 310016, China.

Corresponding author: Zhongheng Zhang

Address: No 3, East Qingchun Road, Hangzhou 310016, Zhejiang Province, China.

Email: [zh_zhang1984@zju.edu.cn](mailto:zh_zhang1984@zju.edu.cn)

Figure S1. Missing rate for clinical and laboratory variables extracted from the database. Variables with missing rate greater than 40% were excluded from analysis.

Table S1 latent profile analysis restricting to patients admitted after the year 2008

|  |  |  |  |  |  | No. of patients in each latent profile | | | | | | |  |
| --- | --- | --- | --- | --- | --- | --- | --- | --- | --- | --- | --- | --- | --- |
| No. of classes | AIC | CAIC | BIC | SABIC | entropy | 1 | 2 | 3 | 4 | 5 | 6 | 7 | P |
| 2 | 1077883.554 | 1078482.373 | 1078406.373 | 1078164.862 | 0.975 | 6209 ( 86 ) | 972 ( 14 ) |  |  |  |  |  | 0.001 |
| 3 | 1073144.556 | 1073948.234 | 1073846.234 | 1073522.101 | 0.958 | 736 ( 10 ) | 5572 ( 78 ) | 873 ( 12 ) |  |  |  |  | 0.001 |
| 4 | 1070750.624 | 1071759.161 | 1071631.161 | 1071224.405 | 0.945 | 514 ( 7 ) | 748 ( 10 ) | 5089 ( 71 ) | 830 ( 12 ) |  |  |  | 0.001 |
| 5 | 1070447.235 | 1071660.631 | 1071506.631 | 1071017.254 | 0.847 | 454 ( 6 ) | 2036 ( 28 ) | 3509 ( 49 ) | 682 ( 9 ) | 500 ( 7 ) |  |  | 0.061 |
| 6 | 1063315.807 | 1064734.062 | 1064554.062 | 1063982.062 | 0.888 | 452 ( 6 ) | 1408 ( 20 ) | 4016 ( 56 ) | 703 ( 10 ) | 493 ( 7 ) | 109 ( 2 ) |  | 0.061 |
| 7 | 1059184.262 | 1060807.376 | 1060601.376 | 1059946.755 | 0.883 | 431 ( 6 ) | 1317 ( 18 ) | 557 ( 8 ) | 3614 ( 50 ) | 680 ( 9 ) | 475 ( 7 ) | 107 ( 1 ) | 0.082 |

Note: * p value was reported for the bootstrap likelihood ratio test comparing current model (k class) to the model with k-1 class.

Abbreviations: AIC: Akaike Information Criterion; CAIC: consistent Akaike Information Criterion; BIC: Bayesian information criteria; SABIC: sample size adjusted Bayesian information criteria.

Table S2 latent profile analysis restricting to patients admitted before the year 2008

|  |  |  |  |  |  | No. of patients in each latent profile | | | | | | |  |
| --- | --- | --- | --- | --- | --- | --- | --- | --- | --- | --- | --- | --- | --- |
| No. of classes | AIC | CAIC | BIC | SABIC | entropy | 1 | 2 | 3 | 4 | 5 | 6 | 7 | P |
| 2 | 1077880.317 | 1078479.136 | 1078403.136 | 1078161.625 | 0.977 | 6244 ( 87 ) | 937 ( 13 ) |  |  |  |  |  | 0.001 |
| 3 | 1073144.306 | 1073947.984 | 1073845.984 | 1073521.85 | 0.958 | 5572 ( 78 ) | 874 ( 12 ) | 735 ( 10 ) |  |  |  |  | 0.001 |
| 4 | 1068900.93 | 1069909.467 | 1069781.467 | 1069374.712 | 0.963 | 5578 ( 78 ) | 433 ( 6 ) | 440 ( 6 ) | 730 ( 10 ) |  |  |  | 0.001 |
| 5 | 1066804.864 | 1068018.26 | 1067864.26 | 1067374.883 | 0.887 | 4238 ( 59 ) | 393 ( 5 ) | 447 ( 6 ) | 1514 ( 21 ) | 589 ( 8 ) |  |  | 0.071 |
| 6 | 1063609.879 | 1065028.134 | 1064848.134 | 1064276.134 | 0.884 | 3866 ( 54 ) | 290 ( 4 ) | 485 ( 7 ) | 1563 ( 22 ) | 427 ( 6 ) | 550 ( 8 ) |  | 0.051 |
| 7 | 1057261.953 | 1058885.067 | 1058679.067 | 1058024.446 | 0.905 | 1167 ( 16 ) | 100 ( 1 ) | 4214 ( 59 ) | 466 ( 6 ) | 309 ( 4 ) | 364 ( 5 ) | 561 ( 8 ) | 0.072 |

Note: * p value was reported for the bootstrap likelihood ratio test comparing current model (k class) to the model with k-1 class.

Abbreviations: AIC: Akaike Information Criterion; CAIC: consistent Akaike Information Criterion; BIC: Bayesian information criteria; SABIC: sample size adjusted Bayesian information criteria.

Table S3 sensitivity analysis restricting to patients who did not receive heparin

|  |  |  |  |  |  | No. of patients in each latent profile | | | | | | |  |
| --- | --- | --- | --- | --- | --- | --- | --- | --- | --- | --- | --- | --- | --- |
| No. of classes | AIC | CAIC | BIC | SABIC | entropy | 1 | 2 | 3 | 4 | 5 | 6 | 7 | P |
| 2 | 1619834.34 | 1620463.865 | 1620387.865 | 1620146.347 | 0.977 | 9251 ( 86 ) | 1505 ( 14 ) |  |  |  |  |  | 0.001 |
| 3 | 1613020.581 | 1613865.469 | 1613763.469 | 1613439.326 | 0.96 | 973 ( 9 ) | 1349 ( 13 ) | 8434 ( 78 ) |  |  |  |  | 0.001 |
| 4 | 1606590.284 | 1607650.536 | 1607522.536 | 1607115.769 | 0.957 | 1208 ( 11 ) | 1154 ( 11 ) | 865 ( 8 ) | 7529 ( 70 ) |  |  |  | 0.001 |
| 5 | 1603927.945 | 1605203.56 | 1605049.56 | 1604560.169 | 0.944 | 1132 ( 11 ) | 1116 ( 10 ) | 895 ( 8 ) | 6977 ( 65 ) | 636 ( 6 ) |  |  | 0.051 |
| 6 | 1600177.197 | 1601668.176 | 1601488.176 | 1600916.16 | 0.944 | 1082 ( 10 ) | 963 ( 9 ) | 845 ( 8 ) | 6780 ( 63 ) | 582 ( 5 ) | 504 ( 5 ) |  | 0.001 |
| 7 | 1587454.517 | 1589160.86 | 1588954.86 | 1588300.219 | 0.947 | 1068 ( 10 ) | 423 ( 4 ) | 943 ( 9 ) | 6847 ( 64 ) | 629 ( 6 ) | 630 ( 6 ) | 216 ( 2 ) | 0.082 |

Note: * p value was reported for the bootstrap likelihood ratio test comparing current model (k class) to the model with k-1 class.

Abbreviations: AIC: Akaike Information Criterion; CAIC: consistent Akaike Information Criterion; BIC: Bayesian information criteria; SABIC: sample size adjusted Bayesian information criteria.

Figure S2. Characteristics of latent profile groups by restricting to patients without using heparin. Note: the specific profile number may not be consistent with the main analysis due to random process.

Abbreviations: aPTT: activated partial thrombin time; GCS: Glasgow coma scale; INR: international normalized ratio; BP: blood pressure; RR: respiratory rate; UO: urine output; VR: vasopressor rate; WBC: white blood cell count.

Table S4 Cox regression model to adjust for confounding for the 90-day survival

| Variables | Hazard ratio | Lower limit of 95% CI | Upper limit of 95% CI | p |
| --- | --- | --- | --- | --- |
| Gender (female as reference) | 1.09 | 1.03 | 1.16 | 0.006 |
| Age (with each year increase) | 1.03 | 1.02 | 1.03 | <0.001 |
| SOFA | 1.13 | 1.12 | 1.15 | <0.001 |
| ICU type (CCU as reference) |  |  |  |  |
| CSRU | 0.64 | 0.55 | 0.75 | <0.001 |
| MICU | 1.13 | 1.03 | 1.25 | 0.010 |
| SICU | 1.01 | 0.89 | 1.14 | 0.883 |
| TSICU | 0.89 | 0.77 | 1.03 | 0.117 |
| RRT (yes as reference) | 0.92 | 0.82 | 1.04 | 0.188 |
| Ethnicity (Asian as reference) |  |  |  |  |
| BLACK | 0.82 | 0.65 | 1.03 | 0.085 |
| HISPANIC | 0.83 | 0.63 | 1.11 | 0.216 |
| UNKNOWN | 1.40 | 1.12 | 1.73 | 0.002 |
| WHITE | 1.11 | 0.91 | 1.36 | 0.320 |
| Profile 1 as reference |  |  |  |  |
| Profile 2 | 1.15 | 1.02 | 1.28 | 0.018 |
| Profile 3 | 1.79 | 1.63 | 1.97 | <0.001 |
| Profile 4 | 1.01 | 0.92 | 1.12 | 0.809 |
| Vasopressor use (yes as reference) | 0.86 | 0.79 | 0.93 | <0.001 |

Abbreviations: SOFA: sequential organ failure assessment; RRT: renal replacement therapy; CCU: coronary artery unit; CSRU: cardiac surgery recovery unit; MICU: medical ICU; SICU: surgical ICU; TSICU: Trauma-Neuro ICU.

The 90-day survival information was obtained by linking to the social security database by the database maintainer.

Table S5 choosing the best number of classes by using latent class analysis

|  | log-likelihood | resid. df | BIC | aBIC | cAIC | likelihood-ratio | Entropy | N of classes |
| --- | --- | --- | --- | --- | --- | --- | --- | --- |
| 2 | -486180.6 | 14850 | 973736.2 | 973281.8 | 973879.2 | 684070.0 | 0.76 | 2 |
| 3 | -482284.6 | 14778 | 966636.5 | 965953.3 | 966851.5 | 676278.0 | 0.78 | 3 |
| 4 | -479994.2 | 14706 | 962748.0 | 961835.9 | 963035.0 | 671697.2 | 0.91 | 4 |
| 5 | -478207.2 | 14634 | 959866.2 | 958725.3 | 960225.2 | 668123.1 | 0.83 | 5 |
| 6 | -476727.0 | 14562 | 957598.2 | 956228.6 | 958029.2 | 665162.8 | 0.78 | 6 |
| 7 | -475301.4 | 14490 | 955439.2 | 953840.8 | 955942.2 | 662311.5 | 0.81 | 7 |
| 8 | -474244.9 | 14418 | 954018.6 | 952191.3 | 954593.6 | 660198.6 | 0.80 | 8 |
| 9 | -473307.8 | 14346 | 952836.6 | 950780.5 | 953483.6 | 658324.3 | 0.80 | 9 |
| 10 | -472556.2 | 14274 | 952025.8 | 949740.9 | 952744.8 | 656821.2 | 0.80 | 10 |

Abbreviations: AIC: Akaike Information Criterion; cAIC: consistent Akaike Information Criterion; BIC: Bayesian information criteria; aBIC: adjusted Bayesian information criteria.

File S1

ICD-9 codes for infection:

|  | WITH infection_group AS |
| --- | --- |
|  | ( |
|  | SELECT subject_id, hadm_id, |
|  | CASE |
|  | WHEN substring(icd9_code,1,3) IN ('001','002','003','004','005','008', |
|  | '009','010','011','012','013','014','015','016','017','018', |
|  | '020','021','022','023','024','025','026','027','030','031', |
|  | '032','033','034','035','036','037','038','039','040','041', |
|  | '090','091','092','093','094','095','096','097','098','100', |
|  | '101','102','103','104','110','111','112','114','115','116', |
|  | '117','118','320','322','324','325','420','421','451','461', |
|  | '462','463','464','465','481','482','485','486','494','510', |
|  | '513','540','541','542','566','567','590','597','601','614', |
|  | '615','616','681','682','683','686','730') THEN 1 |
|  | WHEN substring(icd9_code,1,4) IN ('5695','5720','5721','5750','5990','7110', |
|  | '7907','9966','9985','9993') THEN 1 |
|  | WHEN substring(icd9_code,1,5) IN ('49121','56201','56203','56211','56213', |
|  | '56983') THEN 1 |
|  | ELSE 0 END AS infection |
|  | FROM diagnoses_icd |
|  | ), |
